# A Retrospective Study on Mandibular Reconstruction Following Excision of Canine Acanthomatous Ameloblastoma

**DOI:** 10.3389/fvets.2022.900031

**Published:** 2022-05-11

**Authors:** Anson J. Tsugawa, Boaz Arzi, Natalia Vapniarsky, Frank J. M. Verstraete

**Affiliations:** ^1^School of Veterinary Medicine, William R. Pritchard Veterinary Medical Teaching Hospital, University of California, Davis, Davis, CA, United States; ^2^Department of Surgical and Radiological Sciences, School of Veterinary Medicine, University of California, Davis, Davis, CA, United States; ^3^School of Veterinary Medicine, Veterinary Institute for Regenerative Cures, University of California, Davis, Davis, CA, United States; ^4^Department of Pathology, Microbiology and Immunology, School of Veterinary Medicine, University of California, Davis, Davis, CA, United States

**Keywords:** mandible, critical size bone defect, reconstruction, internal fixation, rhBMP-2, regeneration, dog

## Abstract

The successful excision of a locally invasive tumor such as canine acanthomatous ameloblastoma (CAA) typically results in a mandibular contour-derforming, critical-size defect that alters the jaw kinematics, and may affect the patient's quality of life. In this case series, we describe our experience using the regenerative approach of a titanium locking plate and compression resistant matrix infused with rhBMP-2 for the immediate or delayed reconstruction following mandibulectomy for the excision of mandibular CAA in 11 dogs. Surgical planning included computed tomography (CT), with and without contrast, in all cases, and 3D-printed models in four cases. Tumor-free surgical margins were achieved in all dogs. Clinical and diagnostic imaging follow-up (mean, 23.1 months) were performed in-person (11 cases) and with CT/cone-beam computed tomography in most cases, with standard radiography (3 cases) and telemedicine being utilized in 5 cases. At 2 weeks postoperatively, hard tissue was palpable at the defect. Follow-up imaging at 1 month postoperatively revealed evidence of bridging new bone with a heterogeneous appearance, that remodeled over 3–6 months to bone of a similar size, shape and trabecular pattern as native bone. Histological evaluation of regenerated bone was available in two cases, and was supportive of our clinical and imaging findings of normal remodeled bone. Clinically, all dogs returned to a normal lifestyle, rapidly resumed eating and drinking, and exhibited normal occlusion. Complications included wound dehiscence in one dog and self-limiting exuberant bone formation in two dogs. Tumor regrowth, failure of the implant or fracture of the regenerated bone were not observed. We conclude that the mandibular reconstruction using a regenerative approach is safe, feasible, and results in restoration of mandibular contour in dogs following segmental and bilateral rostral mandibulectomy for benign but invasive oral tumors such as CAA.

## Introduction

Mandibulectomy in dogs may be performed to excise oral tumors, treat chronic infection (i.e., osteomyelitis and osteoradionecrosis) and as a salvage procedure following severe trauma. Two commonly performed partial mandibulectomy techniques are segmental mandibulectomy, where an entire section or vertical height of the mandible has been excised, and bilateral rostral mandibulectomy, where both rostral mandibles up to the level of the mandibular second premolar teeth (or beyond) are excised. Following segmental and bilateral rostral mandibulectomy, dogs are forced to adapt to a “new normal” occlusion and may suffer substantial functional consequences attributable to the loss of mandibular continuity, such as challenges with food prehension. The drift of the intact mandible that occurs following segmental mandibulectomy may also result in traumatic occlusion of the canine teeth against the hard palate and/or the lip (1). Because the dog's temporomandibular joint (TMJ) is normally largely hinge-like, with limited side–side movement, dogs with mandibular drift may suffer a substantial alteration to the mandibular kinematics, which may disrupt TMJ congruity, and may result in secondary degenerative changes to the joints (2). The other complications of mandibulectomy include atrophic changes to the muscles of mastication due to muscular imbalance, and more specific to the brachygnathism imparted by bilateral rostral mandibulectomy, desiccation of the exposed rostral portion of the tongue and moist dermatitis of the cervical skin due to lack of containment of saliva.

Canine acanthomatous ameloblastoma (CAA) is a common odontogenic tumor and subtype of ameloblastoma of predominantly (70%) the mandible, and in particular the rostral mandible, of larger breed adult dogs that is commonly surgically treated by mandibulectomy (3). The biologic behavior of CAA is variable in growth rate and bone destruction. Computed tomographic (CT) imaging, with and without contrast, is considered the diagnostic imaging modality of choice for assessing the severity of bone lysis, which is associated with predominant tumor location (extraosseous or intraosseous), and is integral to surgical planning (4, 5). Canine acanthomatous ameloblastoma is considered a benign tumor because it does not metastasize, but it often behaves aggressively, destroying the underlying bone and requiring extensive mandibular excision (contour-deforming) to achieve a cure. The recurrence rates of CAA are low when clean surgical margins are achieved; however, the scientific definition of the appropriate surgical margins for CAA are less straightforward. Wide surgical excision, defined as 10 mm margins, has been the generally accepted guideline for the excision of CAA. Dogs with successfully excised benign tumors such as CAA, with critical-sized mandibular defects (i.e., osseous defects between 15 and 50 mm that will not heal in the dog's lifetime) are suitable candidates for reconstruction since they have a good long-term prognosis and their lifespan is not threatened by future risk of metastasis (6).

Reconstruction of mandibular critical-sized defects may be performed immediately following mandibulectomy (primary) or delayed (secondary) when the dog's surgical wound bed has healed, and tumor-free margins have been confirmed. The ideal timing, and for which cases mandibular reconstruction should be performed remains controversial (7). A similar regenerative technique to the one reported here (8–10) for mandibular reconstruction was previously reported in people and demonstrated a reduced quality and quantity of regenerated bone with reconstructions that were performed delayed compared to immediately reconstructed bone defects (11). With immediate reconstruction, exposure of the graft and plate to the oral cavity have long been associated with greater graft resorption, infection rates, and pain (12). More recently, the rigidity of the fixation, tension-free closure and achievement of a water-tight seal with the soft tissue closure, have been shown to play a bigger role in overcoming these complications rather than timing of the reconstruction (13). Critical-sized defects may not be amenable to reconstruction using particulate or non-vascularized grafts, and in humans, critical-sized defects of the mandible are typically reconstructed using microvascular techniques with free-fibular grafts. Microvascular surgery requires advanced training and expertise to perform, as well as specialized equipment (operating microscope) and instruments, that are currently not readily available in veterinary medicine. Therefore, in our experience, mandibular reconstruction using a regenerative approach is favored in dogs.

The use of bone morphogenic protein (BMP), specifically recombinant human BMP2 (rhBMP-2), also referenced by its generic name, dibotermin alfa, derived from genetically engineered Chinese hamster ovary cell lines, has shown substantial benefit as a bioactive compound for bone regeneration avoiding the morbidity incurred with a separate donor site for harvesting autogenous bone grafts (9, 14, 15). The BMPs are the members of the transforming growth factor β (TGF-β) growth factor superfamily of proteins. The BMP−2, −4, −6, and −7 have osteoinductive properties, and play an important role in the signaling of the cascade of events responsible for the differentiation of mesenchymal stem cells into osteoblasts. The rhBMP-2 is used in combination with an absorbable carrier that maintains the appropriate concentration of rhBMP-2, prevents heterotopic bone formation, imparts early mechanical support, and serves as a scaffold for osteogenesis. Earlier reports on the use of rhBMP-2 infused into a compression resistant matrix (CRM) in dogs (16–18) reported bone regeneration in critical-size mandibular defetcs following mandibulectomy or gun shot injuries. The technique was later modified to include the use of a single titanium reconstruction plate and locking screws, and shown to successfully restore mandibular continuity and kinematics (8–10, 19).

The objective of this retrospective study was to report on the clinical outcome of a regenerative reconstruction technique for the restoration of continuity following segmental and bilateral rostral mandibulectomy in dogs treated for CAA.

## Materials and Methods

### Case Inclusion

A search of the medical records database of the William R. Pritchard Veterinary Medical Teaching Hospital (VMTH), University of California, Davis, was conducted to identify dogs that underwent immediate (primary) or delayed (secondary) reconstruction following wide surgical excision (defined as a minimum of 10-mm margins), segmental or bilateral rostral mandibulectomy, to treat CAA during a 11-year period (2011–2022). This search was further refined to include only cases with complete medical records, clinical follow-up of at least 2 months and histopathologically confirmed tumor-free surgical margins. Preoperative diagnostic imaging was performed by conventional CT (LightSpeed 16, GE Healthcare, Milwaukee, WI) with and without contrast. Postoperative and subsequent follow-up imaging were performd using a CT or cone-beam computed tomography (CBCT) (NewTom, 5G, Verona, Italy) (Table 1). In selected cases, digital radiography (RapidStudy EDR6, Eklin Medical Systems, Sunnyvale, CA) was also used.

**Table 1:** Summary data for 11 dogs that received segmental and bilateral rostral mandibulectomy for CAA and reconstruction using a regenerative approach.

**Dog**	**Age (years)**	**Sex**	**Breed**	**Weight at surgery** **(kg)**	**Tumor location**	**Mandibulectomy**	**Defect size (cm)**	**Timing of reconstruction**	**Complications**	**Duration of Follow-up (months)**
1	8	MC	Australian shepherd	25.0	Right mandibular first molar	Segmental	4.0	Immediate		70
2	9	MC	mixed breed	35.0	Right mandibular first molar	Segmental	5.0	Immediate		14
3	9	MC	golden retriever	25.0	Right mandibular third molar	Segmental	5.0	Immediate		2
4	5	FS	Chow Chow	22.0	Left mandibular fourth premolar	Segmental	5.0	Delayed		50
5	8	MC	German shepherd	40.0	Left mandibular molars	Segmental	5.5	Immediate	Bone dense mass at caudal aspect of mandibulectomy site, histology revealed bony proliferation and remodeling	6
6	8	FS	Labrador retriever	30.7	Left mandibular fourth premolar	Segmental	4.5	Immediate	Exuberant reaction to rhBMP-2	52
7	7	MC	Labrador retriever	33.5	Left mandibular first and second molars	Segmental	4.5	Immediate		2
8	7	MC	pit bull terrier	31.0	Left mandibular first molar	Segmental	4.0	Immediate		24
9	3	M	mastiff	64.5	Left mandibular canine to right mandibular canine	Bilateral rostral	5.5	Immediate	Partial dehiscence of the left side of the surgical site	18
10	12	FS	Labrador retriever	32	Left mandibular incisors to mandibular canine	Bilateral rostral	5.5	Delayed		6
11	11	MC	mixed breed	22.1	Right mandibular incisors	Bilateral rostral	5.0	Delayed		10

### Medical Records Review

The data on signalment (age, reproductive status, and breed) and physical examination (body weight, tumor size and location) were collected. The information was obtained from the medical records including histopathology results, diagnostic imaging (intraoral radiographs, CT, and tridimensional 3D-printed skull models), surgical technique, and complications. Objective outcome/follow-up information was obtained from review of the medical records, including the dog's post-reconstruction occlusion, and subjective information related to masticatory function (ability to prehend food/types of food), and overall impressions related to return of function, general disposition, and comfort during the postoperative period.

### Diagnostic Imaging

Digital radiographs of the mandibles and CT studies (transverse, 0.625-mm slice thickness, with and without contrast) that were obtained immediately following reconstruction, and at short (1–2 months), medium (3–6 months) and long-term (>6 months) follow-up examinations were reviewed for evidence of new bone formation, integration of the implant material with the native mandibular bone, opacity, and margin character of the implant material, and for the presence of exuberant bone. Screw placement with respect to their proximity to the mandibular tooth roots and mandibular canal of the mandible was evaluated for all dogs.

### 3D Model Printing

For dog 4, where segmental mandibulectomy was performed elsewhere, and for the three bilateral rostral mandibulectomy patients (dogs 9, 10, and 11), 3D-printed models (Polyjet Printer, Stratasys, Rehovot, Israel) were available for review, that had been used for surgical planning and for the adaptation and recontouring of the 2.4-/3.0-mm titanium locking reconstruction plates (Synthes® Maxillofacial, Paoli, PA, USA).

### Surgical Technique

#### Mandibulectomy

For all immediate reconstruction cases, the dogs were intubated normograde prior to the mandibulectomy. Delayed reconstruction cases were intubated by pharyngotomy. The procedures were performed in a combination of dorsal and ventral recumbency. The surgical margins were defined as 10 mm (or more, if a tooth was identified within the measured margins), were measured with a ruler and marked using a surgical marking pen based on the gross assessment of the tumor borders and measurements from the transverse CT and 3D volume rendered images. A detailed presentation of the mandibulectomy technique is beyond the scope of this study and is available elsewhere (20). Specifically, for the delayed reconstruction, closure of the mandibulectomy site was performed with future plate accommodation in mind (i.e., preserving excess skin rather than excising it).

### Reconstruction

#### Reconstruction of Segmental Mandibulectomy

A combination of extraoral and intraoral approaches were used as previously described (9). Briefly, after the surgical margins were delineated with a marking pen, with the dog in dorsal recumbency, a mandibular plate (2.4-/3.0-mm mandibular locking reconstruction plate, Synthes®, Maxillofacial, Paoli, PA, USA) was pre-contoured along the ventrolateral aspect of the mandible prior to the mandibulectomy, and secured to the mandible (ventral to the roots of the teeth) using a minimum of three 3-mm locking screws on each end of the plate. The plate and screws were removed, the dog was positioned in ventral recumbency, and the mandibulectomy was completed via an intraoral approach. Following intraoral closure, the dog was returned to dorsal recumbency and, through an extraoral approach, the pre-contoured plate was adjusted and adapted to the bone using bone-holding forceps, and the locking screws were replaced into the sites made prior to the mandibulectomy. The surgical site was copiously irrigated with sterile 0.9% saline and the CRM (sized to a snug fit between the bone ends), infused with 0.5% rhBMP-2 at a soak volume of 50%, was implanted. The CRM was circumferentially secured using absorbable monofilament suture, and the surrounding soft tissues were used to form a soft tissue envelope around the implant. Care was taken to avoid fenestration of the oral mucosa into the oral cavity and stretch of the soft tissues over the plate. The subcutaneous tissues and skin were closed in a routine fashion.

#### Reconstruction of Rostral Mandibulectomy

Reconstruction of bilateral rostral mandibulectomy was previously described (8). Briefly, specific to the rostral mandibular reconstruction procedure, a vinyl polysiloxane putty (3M ESPE, St. Paul, MN, USA) bite registration impression of the caudal teeth occlusion (fourth mandibular premolar teeth to the mandibular molar teeth) was obtained prior to performing the mandibulectomy to accurately maintain and record the dog's preoperative occlusion. Using the bite registration and a scale 3D-printed skull model based off the CT images, the surgical procedure was rehearsed prior to surgery, and the mandibular reconstruction plates were pre-bent into a horseshoe shape that extended caudally to the level of the mandibular first molar teeth. For the delayed reconstructions, the soft tissue closure was as described above, and for the interim period, the pre-contoured plate and screws were not replaced to control the bone ends and/or other implant inserted into the defect for the purpose of space maintenance. Four weeks later the reconstructive surgery was performed, the previously obtained bite registration was positioned in the mouth to recapture the presurgical occlusion, the patient positioned in dorsal recumbency, and the surgical approach was extraoral through a single midline incision. Final adjustments and adaptation of the pre-bent reconstruction plate to the native mandibular bone were made as described above for the segmental mandibulectomy procedure, and the plate was secured using at least three 3-mm titanium locking screws in each side. The CRM with rhBMP-2 was placed as for the segmental mandibulectomy reconstruction, and closure of the subcutaneous tissues and skin was routine.

#### Postoperative Care

Dogs were offered soft food for 2 weeks following surgery and were administered amoxicillin/clavulanic acid 20 mg/kg by mouth twice daily for 2 weeks postoperatively. Postoperative pain management consisted of a combination of a non-steroidal anti-inflammatory drug (except in the patient with IRIS stage III renal disease) and an opioid analgesic for 7–14 days.

#### Histopathology

For two dogs, histopathology of the regenerated bone was available for review. In dog 5, the biopsy of a mild exuberant bone reaction was obtained 1 month postoperatively. In the second dog (dog 10), post-mortem biopsies of the regenerated bone were obtained 6 months postoperative. The death of the dog was for reasons unrelated to the report. Bone biopsies were obtained using an osteotome and mallet and were formalin fixed. The specimens were decalcified, sectioned at 5 μm and stained with H&E according to standard procedure. All histological specimens were assessed by a veterinary pathologist (NV). Fresh samples from dog 10 were fixed in 2.5% glutaraldehyde and cacodylate buffer (Sigma, St. Louis, MO, USA) for 96 h at 4°C for scanning electron microscopy (SEM). Following dehydration, using increasing ethanol concentrations, the specimens were exposed to liquid nitrogen and cryofractured. The cryofractured specimens were then critical point dried, spatter coated with gold, and mounted. The sections were viewed on a Philips XL30 TMP SEM and images were captured at 15,000 × magnification. Image J software (National Institutes of Health) was used to assess fiber thickness and alignment in different anatomical regions.

## Results

Eleven client-owned dogs were identified that fit the search criteria in the medical records database. The dogs were between 3 and 12-years of age (mean age, 7.9 years) and weighed 22.0–64.5 kg (mean body weight, 32.6 kg) at the time of reconstructive surgery. The dogs were from the following breeds: Australian shepherd (*n* = 1), chow chow (*n* = 1), German shepherd (*n* = 1), golden retriever (*n* = 1), Labrador retriever (*n* = 3), mastiff (*n* = 1), mixed breed (*n* = 2), and pit bull terrier (*n* = 1). The dogs consisted of seven castrated males, three spayed females, and one intact male.

### Mandibulectomy

Eight dogs had a segmental mandibulectomy performed, and three dogs had a bilateral rostral mandibulectomy performed. The tumor locations for the segmental mandibulectomy dogs were right mandibular first molar tooth (*n* = 2), right mandibular third molar tooth (*n* = 1), right mandibular first and second molar teeth (*n* = 1), left mandibular fourth premolar tooth (*n* = 2), left mandibular first molar tooth (*n* = *1*), left mandibular molar teeth (*n* = *1*). The tumor locations for the rostral mandibulectomy dogs were mandibular incisor teeth, left mandibular first incisor to canine teeth, and left mandibular canine to right mandibular canine teeth (*n* = 1 for each location). The segmental defect sizes were 40–55 mm (mean, 47 mm) and for the rostral mandibulectomy dogs, 50–55 mm (mean, 53 mm).

### Segmental Mandibular Reconstruction

**Figure 1:**
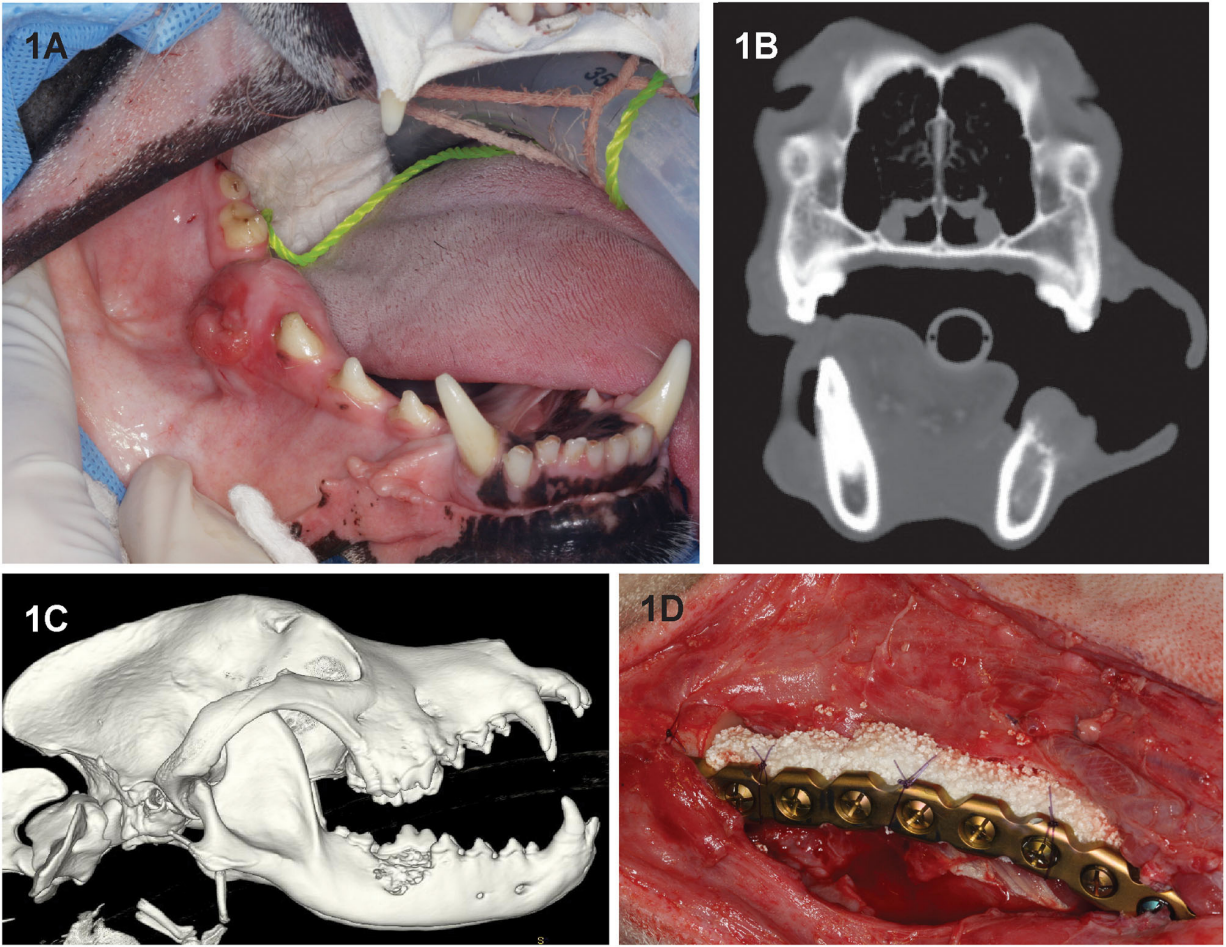
Clinical image **(A)** of dog 2 prior to segmental mandibulectomy of histologically confirmed CAA at the level of right mandibular first molar tooth. **(B)** Sagittal-plane and **(C)** lateral view 3D volume rendering CT images of the same dog. Note that the neoplastic process extends almost to the ventral border of the mandibular canal and exhibits an osteodestructive pattern characteristic of CAA involving the dorsal half of the mandible. **(D)** Intraoperative image following mandibulectomy, replacement of the previously contoured reconstruction plate and insertion of rhBMP-2-soaked CRM implant into the defect **(D)**. The implant was secured to the plate using circumferentially placed poliglecaprone-25 (Monocryl, Ethicon, Somerville, NJ) suture.

Seven dogs had an immediate (primary) reconstruction (Figure 1) performed, and one dog (dog 4) had the mandibulectomy procedure performed elsewhere 70 days before the reconstruction was performed. Follow-up was 2–70 months (mean, 27.5 months).

### Rostral Mandibular Reconstruction

Dog 9 had an immediate (primary) reconstruction performed, and dogs 10 and 11 had delayed (secondary) reconstruction performed. For the delayed reconstruction cases, the length of time between the mandibulectomy and reconstruction procedure was 28–33 days (mean, 30.5 days). Follow-up was 6–18 months (mean, 11.3 months).

### Clinical Assessment and Follow-Up

In-person clinical follow-up was performed on all dogs. Telemedicine (telephone or electronic mail communication) follow-up was available as the last recorded follow-up for dogs 1, 4, 6, 8, and 11. For all dogs, occlusion was deemed appropriate immediately after the surgery and for the duration of follow-up. Other than restriction from heavy chewing for 2–3 months, all dogs experienced a rapid return to normal activity. Within 2 weeks of the reconstruction surgery and throughout the follow-up period, hard tissue was palpable spanning the length of the defect and there was intact soft tissue coverage. Plate exposure or instability were not noted in any case. During the follow-up period all the owners reported that their dogs had good cosmesis and good quality of life, without challenges in mastication or play, and dog 6, who was a service animal, was able to resume all presurgical duties. The owners of dog 8 reported having continued to offer rib bones and engagement with rough rope tugging activities without notable ill-effects on the regenerated bone or construct. Tumor recurrence, construct failure or fractures of the regenerated mandible were not evidenced in any of the dogs.

### Diagnostic Imaging

#### Radiological Evaluation

Follow-up radiographs were available for three dogs (dogs 1, 2, and 3) that received segmental mandibular reconstruction. Increased radiopacity at the implant site was visible starting after 4 weeks. In addition to increasing opacity, bridging new bone formation between the implant and native bone, progressive remodeling of the margins and cortical thickening, were evidenced over time. In dog 2, the 3-month follow-up radiographs revealed an ill-defined radiolucency in the mid mandible with less radiodense fill of the caudal aspect of the implant. Subsequent follow-up radiographs of the same dog at 5 months, 7 months, and 14 months revealed a progressive increase in radiopacity of the implant.

#### Computed Tomographic Evaluation

**Figure 2:**
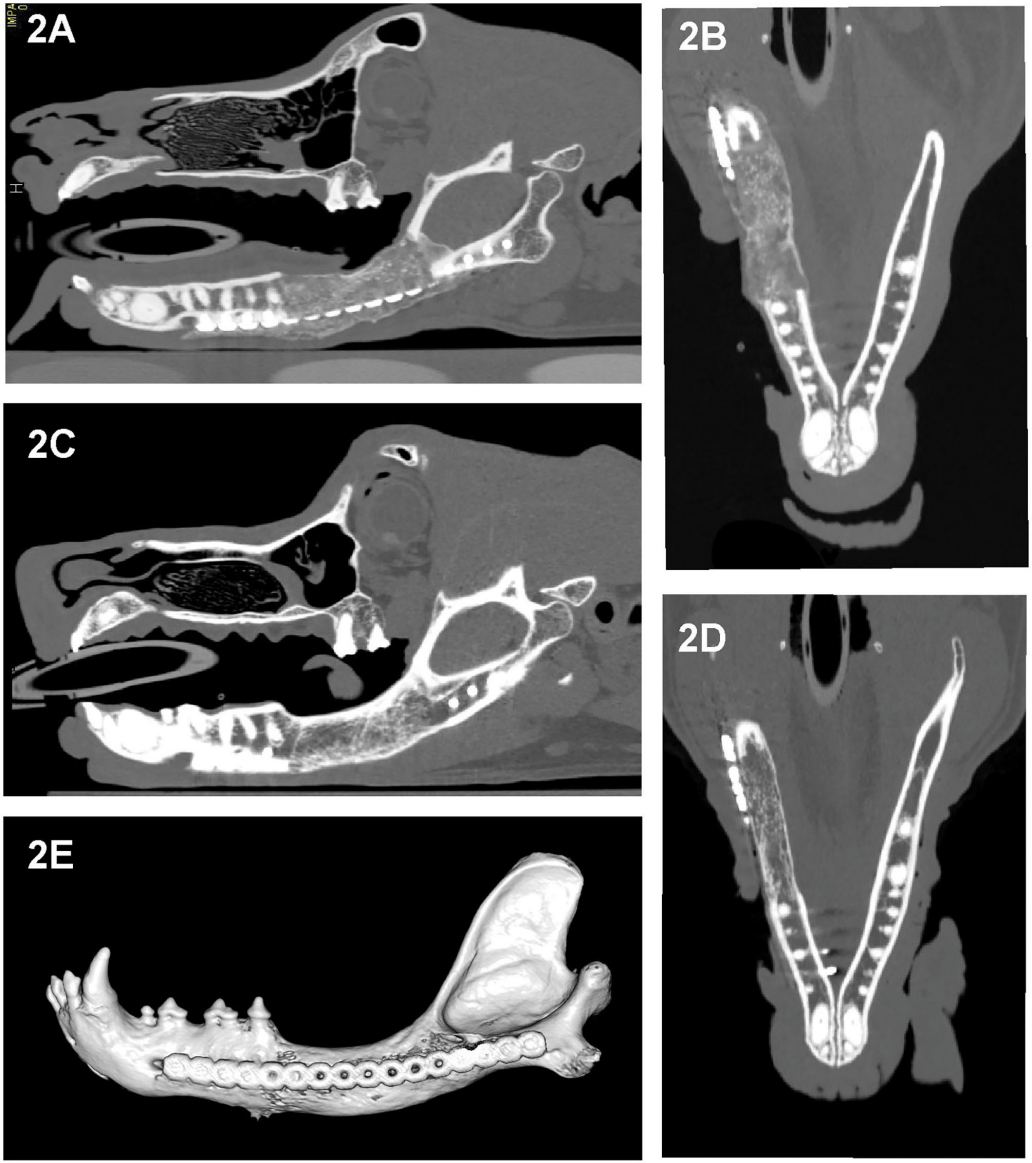
Sagittal- and dorsal-plane reconstructed and 3D volume rendering created from the CT images of dog 5, 1 month **(A,B)** and 6 months **(C–E)** after segmental reconstructive surgery. As early as 1 month postoperatively this dog exhibited bridging new bone formation with implant material replaced by regenerated bone that is expansile and has irregular margins **(A,B)**. At 6 months, the regenerated bone has remodeled, exhibited smoother margins and homogeneity with barely perceptible interface with the native bone and with shape and cancellous pattern similar to the native bone **(C–E)**.

**Figure 3:**
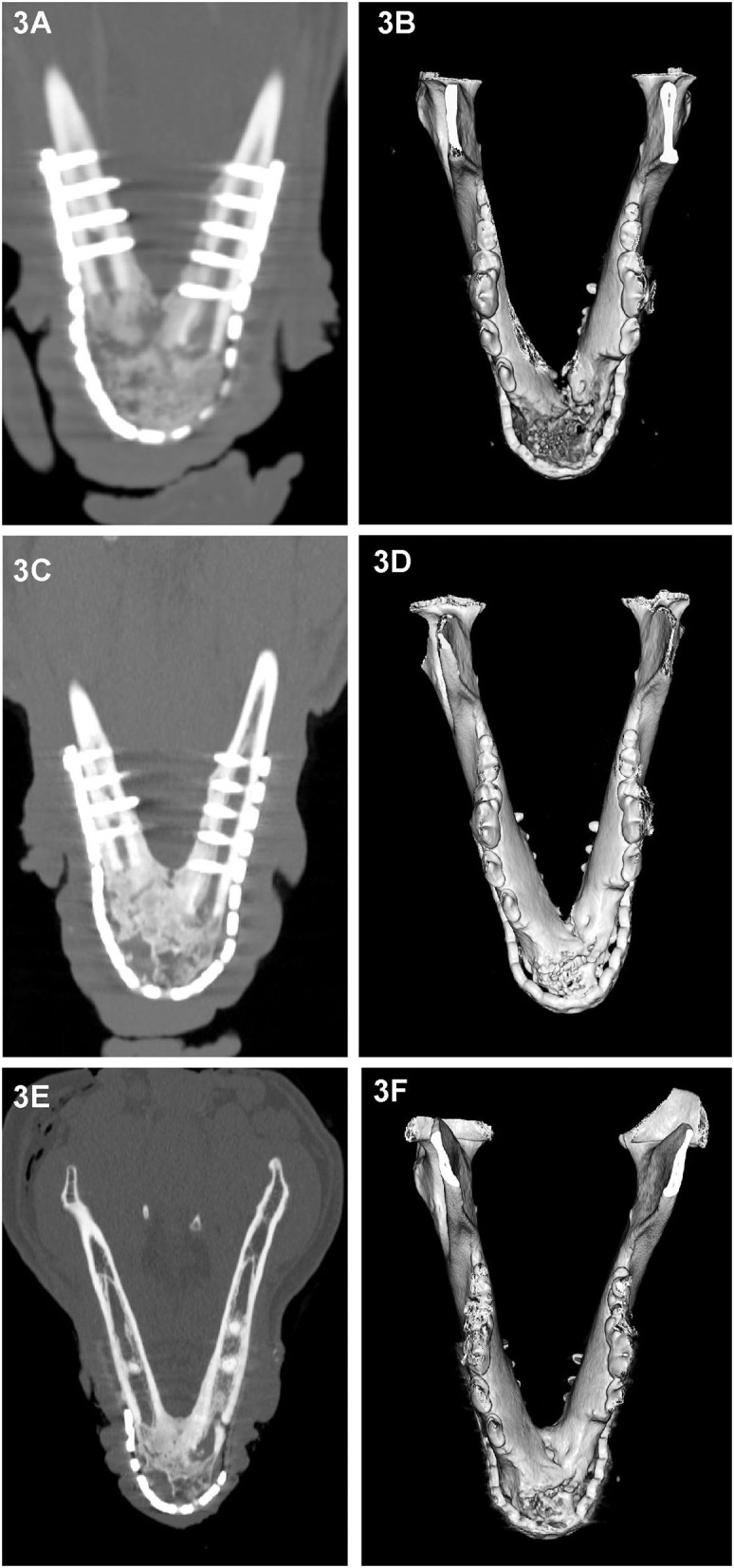
Dorsal-plane reconstructed and 3D volume rendering of CT images of the mandibles of dog at 1 month **(A,B)**, 3 months **(C,D)** and 6 months **(E,F)** after rostral mandibular reconstructive surgery. The right side of the dog is displayed at the left side of each image. The radiolucent gap between the implant material and native mandible that is clearly identifiable on the 1 month dorsal-plane view **(A)** becomes progressively less distinct **(C)** over time and is barely discernible at 6 months **(E)**. Although at 6 months, the regenerated bone in this dog still exhibited heterogeneity, there has been substantial remodeling change as evidenced by the reduction in expansion and cortication of the margins **(A,E)**.

All cases had preoperative conventional CT scans with and without contrast, eight cases had postoperative CT or CBCT scans, and six cases had follow-up CT or CBCT scans that were reviewed. As CBCT is a more emergent technology, earlier performed cases received postoperative CT scans rather than CBCT. Acquisition speed, excellent bone and teeth details and lack of metal artifacts, which are favorable with CBCT, also influenced our decision to use CBCT for postoperative and follow-up scans. Throughout the follow-up period, implant (CRM and rhBMP-2) positioning and appearance remained static, and without radiolucencies. In dog 4 and dog 6, the rostral-most screw was close to the apices of the mandibular canine tooth, and there were no implant-related lucencies. New bone formation was evidenced as early as 1 month on the CT scans (Figure 2A). Over time, as new bone regenerated the remodeled bone became increasingly more homogeneous, the transition between the implant and native bone became less distinct, and the margins of the bridging periosteal new bone formation became progressively smoother (Figures 2A,E). In one rostral mandibular reconstruction case, 19-months follow-up CT demonstrated uniformity throughout the mandible with a trabecular pattern. Starting at 3 months, bone regeneration at the implant site became less expansile and began to follow the model of the implant and shape of the normal mandible. Between 3 and 6 months, normal anatomic structures such as the outline of a mandibular canal-like structure and ventral margin bone became visible. In dog 9, a linear radiolucent line between the left and right sides that resembled the mandibular symphysis became evident at 2 months (Figures 2, 3).

#### Histological Evaluation

**Figure 4:**
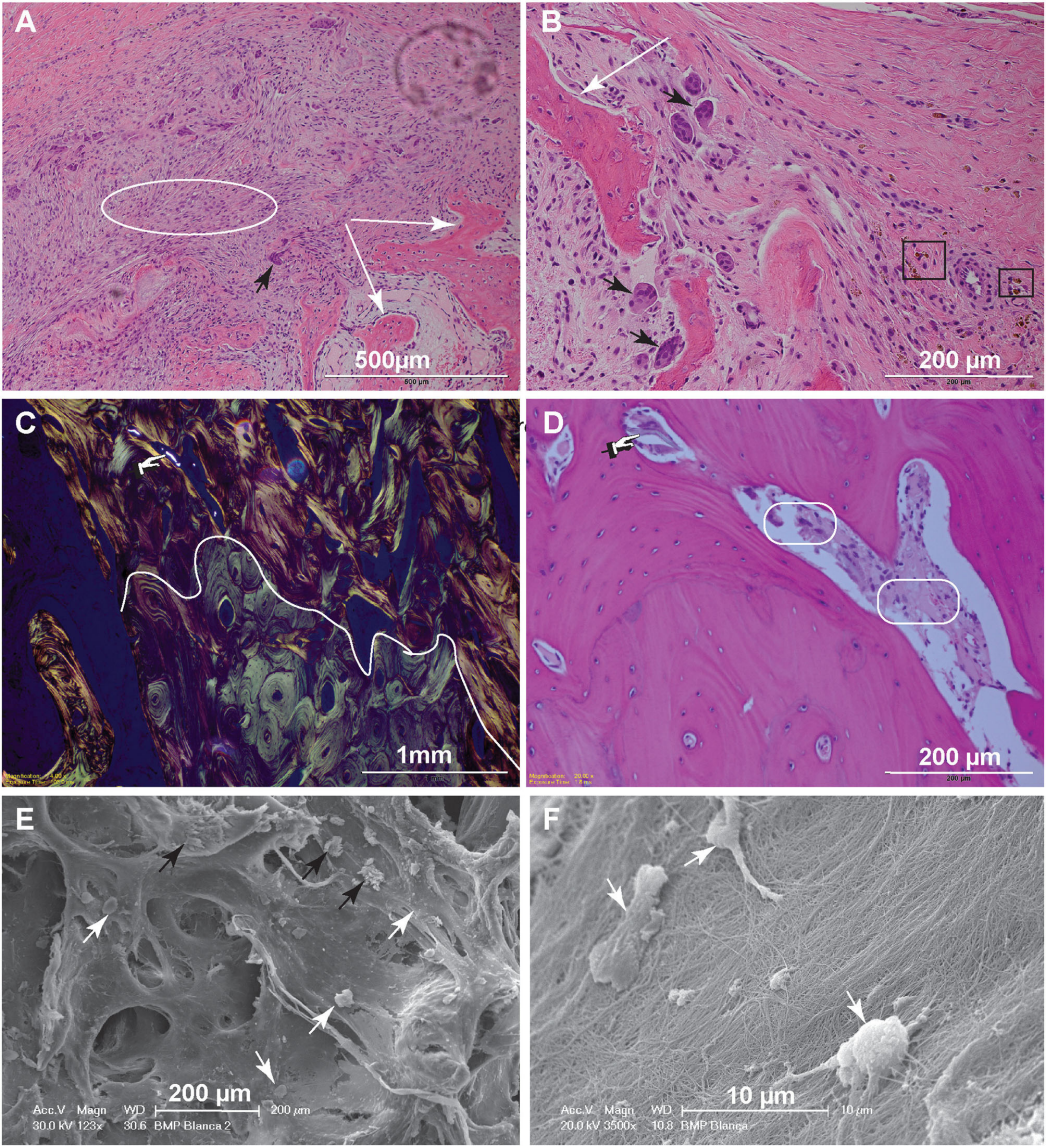
Histopathology and SEM. **(A)** Low magnification image of the biopsy of dog 5, 1-month postoperative. The woven bone trabeculae (white arrows) are outlined by osteoblasts and contain intertrabecular loose fibromatous matrix. Active bone remodeling is evident by the presence of osteoclasts (large multinucleated cells) (black arrow) (bar = 500 μm). **(B)** High magnification image highlighting multiple resorption lacunae occupied by large reactive osteoclasts (black arrows). Hemosiderin–laden macrophages, shown in the black rectangles, indicate historic hemorrhage (bar = 200 μm). **(C)** Low magnification image of the biopsy from dog 10, 6-months postoperatively. This polarized image of bone demonstrates a clear delineation of pre-existing lamellar bone with osteons characterized by circumferential arrangement of collagen fibers (bottom of the curvy line) and immature (woven) bone with collagen fibers arranged in more chaotic pattern (above the curvy line). The refractile material is consistent with remaining CRM (bar = 1 mm). **(D)** High magnification image of woven bone from image **(C)**. Note the lose fibrous connective tissue among the woven bone trabeculae (round corner white rectangles) and inorganic bone scaffold fragment (pointing finger) (bar = 200 μm). **(E)** Scanning electron microscopy demonstrating woven bone with multiple resorption lacunae. The cells represent osteoclasts (black arrows), while more round cells are osteoblasts (white arrows) (bar = 200 μm). **(F)** Higher magnification demonstrates the osteoblasts attached to the collagen fibers. The osteoblasts range in size, 7–10 μm and have multiple cytoplasmic processes (bar = 10 μm).

In dog 5, which exhibited a mild exuberant bone reaction at 1 month postoperatively, the histology revealed woven bone with immature bony trabeculae anastomosing with one another (Figures 4A,B). The intertrabecular spaces were occupied by highly cellular fibro–myxomatous matrix. Cells comprising this matrix were small spindle cells with indistinct borders and small oval nuclei with condensed chromatin. Also, within the fibro–myxomatous matrix there were small islands of osteoid characterized by denser collagenous stroma and osteocytes embedded in it. Near the woven bone trabeculae and osteoid matrix there were numerous large and reactive osteoclasts characterized by multiple nuclei and abundant cytoplasm. These features were interpreted as active bone remodeling.

For dog 10, the biopsies obtained 6 months postoperatively (at post-mortem) revealed mature lamellar bone that closely interfaced woven bone. The interface was traced with polarized light-highlighting collagen bundles that were tightly packed in a concentric pattern in the mature lamellar bone and were less orderly and dense in the areas of woven bone. The bone marrow cavities of the woven bone areas contained loose fibrous connective tissue entrapping spindle fragments of highly refractile material that was often associated with reactive osteoclasts [presumed remnants of hydroxyapatite/tricalcium phosphate (TCP)] (Figures 4C,D).

Scanning electron microscopy further highlighted the cellular players of bone remodeling at an ultrastructural level. These cells represent osteoclasts and osteoblasts present at the bone remodeling interface (Figures 4E,F).

#### Complications

One dog (dog 9) experienced a minor dehiscence that was noticed 11 days following immediate rostral reconstruction. The dehiscence was repaired with débridement and flap revision surgery and was confirmed as healed 14 days later. Two segmental mandibular reconstruction dogs (dog 5 and dog 6) experienced mild exuberant bone formation observed on clinical examination between 1 and 2 weeks following surgery and neither dog exhibited symptoms of pain and/or distress related to the excessive bone formation. The exuberant bone reaction resolved spontaneously within 2 months.

## Discussion

This study evaluated retrospectively 11 dogs that underwent mandibulectomy for the excision of CAA followed by mandibular reconstruction using rhBMP-2 and a CRM. We demonstrated that CAA of the mandibles that required wide excision by means of segmental or bilateral rostral mandibulectomy could be reconstructed with a favorable outcome. Second, imaging follow-up studies demonstrated rapid bone regeneration and remodeling. The timing of reconstruction did not appear to influence the success of the procedure for segmental mandibulectomy patients, but for bilateral rostral mandibulectomy patients, complications related to dehiscence occurred only in the first patient, who had immediate reconstruction, and not the two dogs that had delayed reconstruction. The diagnostic imaging at follow-up correlated with clinical findings of rapid return to normal function and pre-surgical quality of life.

All 11 dogs in this study successfully underwent curative intent wide excision surgery of their mandibular CAA. Wide excision was defined using the traditional guidelines of a 10-mm margin width of normal tissue surrounding the tumor as determined by preoperative assessment of tumor involvement using CT and intraoperative clinical assessment. Although the prognosis and recurrence rate with CAA likely remains low even when narrowly excised (21), due to the regenerative nature of this reconstructive technique, histologically clean margins are necessary to maintain biologic control of such a potent regenerative process that could theoretically result in unwieldy tumor regrowth if not tumor-free. This report informs on the use of regenerative approach to mandibular reconstruction for CAA and is in agreement with the current human literature on the use of this approach for benign tumors (22–24). In all cases reported here, the excision created a critical-sized defect with resultant substantial malocclusion that would have remained without the performed reconstruction. As our understanding of mandibular kinematics in the dog evolves, the severe consequences of the loss in mandibular continuity following mandibulectomy are better understood, and the sequelae of the unrestored mandible is increasingly less acceptable as an outcome (19). Segmental and bilateral rostral mandibulectomy results in mandibular instability that impacts mastication, and recreational activities that require grasping and holding of objects. The deranged mechanical forces that are subsequently transferred to the TMJ have long-term consequences, and painful degenerative changes to the joint may develop. Besides the obvious facial esthetic and social inclusion aspects of leaving the mandible unrestored, humans also suffer from altered phonation and facial mimicry that are not readily appreciated in dogs (25). Without appropriate oral rehabilitation, dogs and humans (26) may suffer malnutrition and weight loss following mandibulectomy. While more conservative options, such as elastic retraining chain (1) and occlusal adjustment, can be used to manage postoperative malocclusion in dogs, neither option restores the continuity of bone, support of the floor of the mouth, bite forces, or kinematics, and does not reduce stress on the muscles of mastication or prevent degenerative changes in the TMJ.

Mandibular reconstruction as previously described (8–10), using locking reconstruction plates and screws combined with rhBMP-2 in a CRM, provided stability, maintained anatomic control of the bone ends, and allowed rapid regeneration of bone that spanned the defect and was clinically functional beginning as early as 1 month postoperatively. The reconstruction procedure used in this case series restored the dogs' relative jaw length and mandibular contour, allowed all 11 dogs to resume a normal quality of life shortly after major jaw surgery, and proved durable (without failure of the construct or fracture of the regenerated bone) over a collective mean follow-up period of nearly 2 years (mean, 23.1 months).

The clinical impressions, radiographic and CT imaging findings were similar and in agreement with previous reports from our group (8–10). As early as 2 weeks post-reconstruction, clinical examination revealed hard tissue that spanned the defect, and both radiographically and on CT, new bone formation was evidenced starting between 1 and 2 months. Between 3 and 6 months imaging revealed bone remodeling. The regenerated bone remodeled, became less expansile with smoother margins, and the initially radiolucent transition gap between the implant and native bone became less distinct. Beyond 6 months the regenerated bone became progressively homogeneous and similar in appearance to the normal mandible with respect to size, shape, cancellous bone pattern, as well as cortication of the ventral surface of the mandible.

All but three of the mandibular reconstruction surgeries were performed immediately following mandibulectomy. Delayed mandibular reconstruction, where the reconstructive portion of the procedure was performed approximately 30 days later, was intentionally performed in the chronologically later rostral mandibular reconstruction cases, dogs 10 and 11, as our experience with this technique evolved, and in only one segmental mandibular reconstruction case, dog 4, that had the mandibulectomy performed at another facility prior to presentation. Following a partial suture line dehiscence seen in dog 9 that received an immediate rostral mandibular reconstruction, timing of the subsequent rostral mandibular reconstruction surgeries was delayed to allow for sufficient healing of the soft tissues before reconstruction. Dog 9 was also a mastiff breed, and the redundant, heavy labial conformation likely contributed to the dehiscence. Dehiscence was not observed in the delayed reconstruction cases, likely due to avoiding the mucosal incision present directly over the plate and the rhBMP-2. Delay of the reconstruction also allowed for maturation of the wound bed (7) and improved asepsis through use of an extraoral incision that was easier to cleanse, and avoided exposure of the internal fixation hardware to the oral flora. Grafts placed through a skin incision are more successful than those placed intraorally (27). A recent 30-year retrospective study in the human medical literature emphasized the presence of a healthy graft soft tissue bed as the primary variable associated with success of large-sized grafts, not the primary tissue diagnosis (benign tumor, osteomyelitis or medication-related osteonecrosis), and reported a high level of success with non-vascularized grafts used for the immediate reconstruction of mandibular defects of up to 6 cm in length (28). In this case series, the timing, immediate or delayed, of mandibular reconstruction did not appear to substantially influence the overall success of the procedures, as all 11 dogs experienced a similar rapid regeneration and remodeling of mandibular bone irrespective of surgical timing. With the limitation of the small sample size of this case series, moving forward, we recommend delayed reconstruction of the rostral mandibles to allow soft tissue healing, of the oral mucosa in particular, so that an intact soft tissue envelope will be available to cover the plate and rhBMP-2 infused CRM. Furthermore, we recommend the use of a single locking reconstruction titanium plate placed in a buccal location, just ventral to the root tips and avoiding a more dorsal position to reduce the chance of plate exposure through the mucosa as noted in earlier reports (16, 18).

Exuberant bone formation was identified in only two segmental mandibulectomy reconstruction cases (dog 5 and dog 6). The only known variable in the regenerative technique was the CRM size that was used (i.e., the protocol for sizing of the CRM was half to three-quarters of the mandibular bone height), and the soak volume of the rhBMP-2 is calculated relative to the size of the CRM (i.e., 50% of the volume of the CRM). Bone formation by rhBMP-2 is both dose- and time-dependent (29), and, given that an identical protocol was used in all dogs, the true cause of the exuberant bone formation remains elusive. The non-painful exuberant bone reaction resolved uneventfully within 2 months and correlated with the reduction in size of the new bone seen on radiographs and CT. Histology of the exuberant bone that was available from dog 5 was consistent with active bone remodeling and confirmed the benign characterization of this reaction. Aside from the acute and correctable complication of surgical site dehiscence and transient, non-painful exuberant bone formation in a small number of patients, the dogs in this study did not suffer any complications related to mandibular reconstruction. Acute orofacial edema is the most common reported complication in humans undergoing mandibular reconstruction with rhBMP-2; that can be made paradoxically worse when rhBMP-2 is used in highly vascularized areas; however, this was not a complication appreciated in the dogs in this series (23, 30). Other known complications of rhBMP-2 such as infection, ectopic bone formation, osteolysis, airway compromise associated with cervical swelling were also not observed (23). The true incidence of these complications in humans, and in dogs as well, are unknown.

Limitations of this study included the small sample size and reliance on telemedicine to achieve long-term follow-up in five cases where in-person follow-up was not possible (i.e., owner moved). The retrospective nature of this study imparted its own limitations. Follow-up CT imaging was not available at every visit, and skull radiographs were used as a financially more affordable alternative at the early stages of this work. Histology of the regenerated bone was available in only two cases where it was indicated due to the presence of exuberant bone or available due to the unrelated death of the dog.

In conclusion, CAA is a common benign but locally invasive tumor occurring in the mandibles (and maxillas) in dogs that may require extensive mandibular excision. If a mandibular contour-deforming excison is performed without mandibular reconstruction, permanent alteration to their mandibular kinematics will ensue. The regenerative technique used here (i.e., immediate or delayed reconstruction with CRM infused with rhBMP-2 and internal fixation) was proven successful in restoring mandibular continuity following extensive mandibulectomy, and allowed all dogs to resume normal activities and have a normal quality of life. Within a month, all dogs developed palpable, clinically functional bone at the surgical site that (based on radiographic and CT findings) remodeled over the course of 3–6 months to bone of similar size and shape to the surrounding normal mandible. Surgical timing of the reconstructions did not seem to influence overall outcome, although delayed timing was beneficial for bilateral rostral mandibular reconstruction. None of the dogs had tumor recurrence or experienced fracture of the regenerated bone or construct.

## Data Availability Statement

The raw data supporting the conclusions of this article will be made available by the authors, without undue reservation.

## Ethics Statement

Ethical review and approval was not required for the animal study because the study is retrospective in nature and included clinical cases, hence, it is exempt from IACUC requirements. Written informed consent for participation was not obtained from the owners because the study is retrospective in nature and, hence, it is exempt from written informed consent.

## Author Contributions

AT: image analysis, data acquisition, interpretation, drafting of the manuscript, and final approval of the version to be published. NV: histopathology analysis, drafting of the manuscript, and final approval of the version to be published. BA and FV: study concept and design, data interpretation, drafting of the manuscript, and final approval of the version to be published.

## Conflict of Interest

The authors declare that the research was conducted in the absence of any commercial or financial relationships that could be construed as a potential conflict of interest.

## Publisher's Note

All claims expressed in this article are solely those of the authors and do not necessarily represent those of their affiliated organizations, or those of the publisher, the editors and the reviewers. Any product that may be evaluated in this article, or claim that may be made by its manufacturer, is not guaranteed or endorsed by the publisher.
